# MircroRNA Profiles of Early Rice Inflorescence Revealed a Specific miRNA5506 Regulating Development of Floral Organs and Female Megagametophyte in Rice

**DOI:** 10.3390/ijms22126610

**Published:** 2021-06-21

**Authors:** Zhixiong Chen, Yajing Li, Peigang Li, Xiaojie Huang, Mingxin Chen, Jinwen Wu, Lang Wang, Xiangdong Liu, Yajuan Li

**Affiliations:** 1 State Key Laboratory for Conservation and Utilization of Subtropical Agro-Bioresources, South China Agricultural University, Guangzhou 510642, China; chenzx@scau.edu.cn (Z.C.); jwwu@scau.edu.cn (J.W.); wanglan@scau.edu.cn (L.W.); 2 Guangdong Provincial Key Laboratory of Plant Molecular Breeding, South China Agricultural University, Guangzhou 510642, China; 3 Department of Plant Breeding, College of Agriculture, South China Agricultural University, Guangzhou 510642, China; lyj6789@stu.scau.edu.cn (Y.L.); peigangl@ibcas.ac.cn (P.L.); huangxj@stu.scau.edu.cn (X.H.); chenmx18270352877@stu.scau.edu.cn (M.C.); 4 Guangdong Laboratory for Lingnan Modern Agriculture, South China Agricultural University, Guangzhou 510642, China; 5 Center of Experimental Teaching for Common Basic Courses, South China Agricultural University, Guangzhou 510642, China

**Keywords:** rice, floral development, miRNA, embryo sac, female gametophyte

## Abstract

The developmental process of inflorescence and gametophytes is vital for sexual reproduction in rice. Multiple genes and conserved miRNAs have been characterized to regulate the process. The changes of miRNAs expression during the early development of rice inflorescence remain unknown. In this study, the analysis of miRNAs profiles in the early stage of rice inflorescence development identified 671 miRNAs, including 67 known and 44 novel differentially expressed miRNAs (DEMs). Six distinct clusters of miRNAs expression patterns were detected, and Cluster 5 comprised 110 DEMs, including unconserved, rice-specific osa-miR5506. Overexpression of osa-miR5506 caused pleiotropic abnormalities, including over- or under-developed palea, various numbers of floral organs and spikelet indeterminacy. In addition, the defects of ovaries development were frequently characterized by multiple megasporocytes, ovule-free ovary, megasporocyte degenerated and embryo sac degenerated in the transgenic lines. osa-miR5506 targeted REM transcription factor *LOC_Os03g11370*. Summarily, these results demonstrated that rice-specific osa-miR5506 plays an essential role in the regulation of floral organ number, spikelet determinacy and female gametophyte development in rice.

## 1. Introduction

Rice is a staple food for a major population in the world. To meet the sustainable increase of population [1], breeding high-yield rice varieties becomes a goal of modern breeding projects due to the adverse production environment, including changing climate, diminishing water and decreasing land availability [2]. Rice yield is a complicated agronomic trait, which is influenced by the number of fertile tillers and the architecture and development of inflorescence [3]. Rice inflorescence consists of a rachis (the main axis), primary branches, secondary (or higher-order) branches and spikelets. A spikelet is the structural unit of inflorescence and comprises two rudimentary glumes, two empty glumes (also called sterile florets) and one fertile floret. The establishment, activity and transition of apical and axillary meristems contribute to the spikelets differentiation, which determines the number of seeds per panicle. It was demonstrated that genetic and epigenetic factors act together to regulate the inflorescence morphology specification. Multiple genes, such as *Oryza sativa homeobox1* (*OSH1*), *CYTOKININ OXIDASE2* (*OsCKX2*), *DROUGHT AND SALT TOLERANCE* (*DST*), *Aberrant Spikelet Additionally, Panicle1* (*ASP1*), *LAX PANICLE1* (*LAX1*), *OPEN BEAK* (*OPB*), *FLORAL ORGAN NUMBER1* (*FON1*) and *FON2/FON4*, are involved in meristem size and maintenance, initiation and outgrowth of axillary meristems [4,5,6,7,8,9,10]. The conserved ABCDE model is applicable to specify the identity of floral organs in rice, besides the different roles of *OsMADS14*, *OsMADS15*, *OsMADS3* and *OsMADS58* [11]. The common regulatory mechanisms/factors involved in regulating inflorescence development, including CLV-WUS signaling, the auxin and cytokinin pathways, MADS-box genes, exist between monocot rice and dicot *Arabidopsis* [12].

The development of male and female gametophytes is important for sexual reproduction. In rice, the molecular mechanism conferring the male gametophyte development has been extensively studied due partly to the convenience of obtaining tissues and various mutants. Numerous genes involved have been identified and functionally analyzed [13,14]. Megasporogenesis and female megagametophyte genesis occur in the ovule, which is a lateral organ initiated from the terminating floral meristem [15]. Due to ovule tissue embedded in carpel and the subsequent difficulty in harvesting them for studies, only a few genes involved in female gametophyte development have been characterized. The mutation in *MULTIPLE SPOROCYTES1* (*MSP1*) results in an increased number of both male and female sporocytes [16]. In the *meiosis arrested at leptotene1* (*mel1*) mutant, the progression of premeiotic, meiosis and mitosis are arrested, causing the failure of female gametophyte formation [17]. *HOMOLOGOUS PAIRING ABERRATION IN RICE MEIOSIS1 (PAIR1*), *PAIR3*, *RAD51C* and *OsMSH4* are involved in the meiosis of megaspore mother cell [18,19,20,21,22]. The abnormalities of mitotic divisions were observed during megagametogenesis in the mutant *anaphase-promoting complex 6* (*OsAPC6*) and *DEFECT IN EARLY EMBRYO SAC1 (OsDEES1*) RNA interference transgenic plants, respectively [23,24]. Although several key regulatory genes were characterized, the molecular mechanism underlying rice female gametophyte development remains investigated intensively.

As a class of endogenous non-coding RNAs, microRNAs (miRNAs) affect many aspects of plant development by regulating the expression level of target genes [25]. Recently, an increased number of miRNAs has been identified to control the inflorescence development in rice. osa-miR156 downregulates (*SQUAMOSA PROMOTER-BINDING PROTEIN-BOX gene 14* (*OsSPL14*), causing an increase in the number of panicle branches and yield [26,27]. osa-miR408 and osa-miR397 positively regulate the number of panicle branches but negatively target different genes [28,29]. The overexpression of osa-miR172 results in the loss of spikelet determinacy and floral organ abnormalities in rice [30]. The decreased number of spikelets was attributed to the smaller inflorescence meristem and fewer primary branch primordia in transgenic lines overexpressing osa-miR529 [31]. The ectopic expression of osa-miR535 causes more but shorter panicles with fewer primary/secondary branches [32]. Most of the well-characterized miRNAs belong to the conserved family. However, most miRNAs appear to be species-specific and usually present organ- or organ-specific expression in plants [33]. The specific miRNAs involved in floral development are worth further functional analysis to broaden the knowledge of miRNAs-mediated regulation on floral development in rice.

During early rice inflorescence development, the identity of different types of meristems is initiated sequentially and promptly, influencing panicle architecture and the number of spikelets consequently. The gradual changes of protein-coding genes expression were detected during the transition between different meristems of early rice inflorescence, using an RNA-sequencing approach [34]. The profiles of miRNAs expression were revealed at late stages of rice inflorescence development [35,36]. However, the alternation of miRNAs expression has not been carried out during the early stages of rice inflorescence development. In this study, deep sequencing of small RNAs was performed to determine the differential expression levels of miRNAs in the early stage of rice inflorescence, covering the development of rachis, branch and spikelet meristems. The potential targets of miRNAs were involved in multiple metabolic and hormone regulation during inflorescence development. It was also revealed that overexpression of a rice-specific osa-miR5506 caused developmental abnormalities, such as the loss of spikelet determinacy, various floral organs and megagametophyte, in rice. The results broaden the knowledge of the important regulatory roles of rice-specific miRNAs in rice inflorescence and reproduction development.

## 2. Results 

### 2.1. Change of miRNA Population during Early Inflorescence Development in Rice

**Figure 1:**
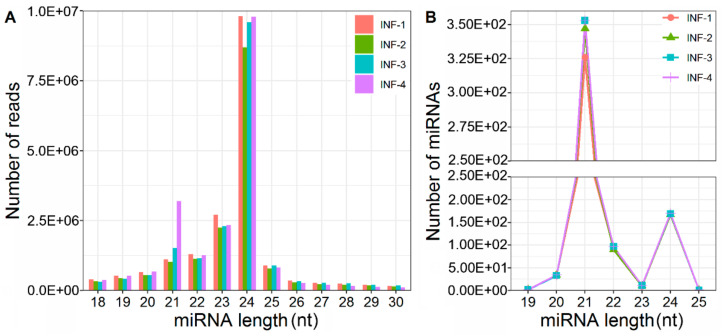
Length distribution of osa-miRNAs of four different libraries of early rice inflorescence development. (**A**), Total reads of osa-miRNAs in different libraries. (**B**), Unique osa-miRNAs in different libraries. INF-1, young inflorescence less than 2 mm length; INF-2, young inflorescence between 2 and 5 mm length; INF-3, young inflorescence between 5 and 10 mm length; INF-4, young inflorescence between 10 and 15 mm length.

In this study, the expression profiles of non-coding miRNAs during the early development of rice inflorescence were conducted. Small-RNA sequencing yielded a total of 248,487,926 raw reads, including 218,606,035 high-quality clean reads. By filtering out the sequences related to rRNAs, tRNAs, snRNAs, snoRNAs or repeat-associated small RNAs, the unannotated reads, ranging from 12,397,583 to 19,037,346 in the number of different libraries, were finally obtained for further miRNAs prediction (Table S1). Furthermore, the clean reads were mapped to mature miRNAs with different nucleotide lengths in *Oryza sativa*, using Bowtie software. Among four different developmental stages of INF-1, INF-2, INF-3 and INF-4, 21–24nt miRNAs are the major population and the highest abundance of reads was found in 24nt miRNAs, followed by 23nt, 21nt and 22nt miRNAs (Figure 1A). Of the unique miRNAs with different-length nucleotides, the 21nt ones were the most abundant in each library and the number of 21nt ones was the greatest in INF-3, compared to INF-1, INF-2 and INF-4 (Figure 1B). It was implied that 21nt miRNAs might play vital roles in early inflorescence development in rice.

With miRDeep2 software, a total of 671 miRNAs, consisting of 559 known and 112 novel ones (Table S2), were identified, after removal of the miRNAs of low abundance with a total number of reads fewer than 10 in twelve samples. A total of 638 miRNAs was common among all four different stages, and only a relatively small fraction of miRNAs could be assumed to represent stage-specific miRNAs. A total of 139 osa-miRNAs were detected at a low expression level with an average of less than 10 transcripts per million (TPM), 231 at a moderate level ranging from 10 to 100 TPM and 301 at a high level of over 100 TPM. Most experimentally verified miRNAs presented a moderate or high expression level in this study. osa-miR156 and osa-miR529 were highly enriched and displayed inversely correlated expression patterns; the former was gradually decreased and the latter increased. osa-miR172a and osa-miR172b showed a converse expression trend. The members of osa-miR2118 family expressed highly and increased gradually from INF-1 to INF-4, while osa-miR2275 families were only detected at high level at INF-4. Moreover, 10 miRNAs were randomly selected for qRT-PCR validation (Figure S1); the expression patterns were largely consistent with the RNA-seq profile, indicating the accuracy of samples used in the present study. Together, these data highlighted the importance of the constant expression of miRNAs during early rice inflorescence development, besides the involvement of stage-specific miRNAs.

### 2.2. Identification of Differentially Expressed miRNAs and Targeted Genes Prediction

With a criterion (|log2(FC)| ≥ 1, FDR ≤ 0.01), we identified 111 differentially expressed miRNAs (DEMs), which accounted for 16.5% of the total miRNAs. Of all DEMs, 67 were known and 44 were novel. It was astonishing that all these DEMs were up-regulated, including 23, 83 and 78 DEMs between two adjacent stages from INF-1 to INF-4, respectively. The 17 members out of the conserved osa-miR2118 family were major among the DEMs. osa-miR5509, osa-miR5516a and osa-miR5516b were expressed differentially among three successive stages. The 34 conserved DEMs were expressed at relatively higher level, compared to the 33 unconserved ones (Figure S2).

**Figure 2:**
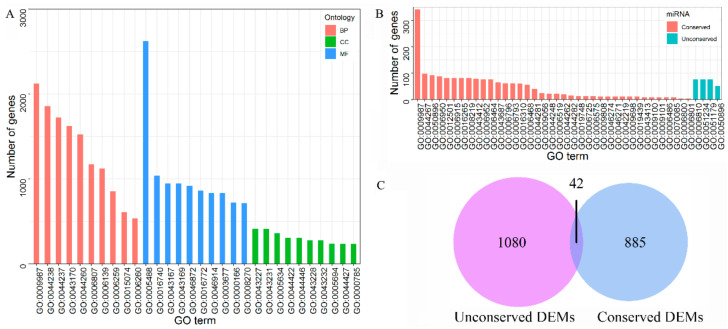
GO analysis of genes targeted by differentially expressed miRNAs (DEMs). (**A**), ten GO terms mostly enriched in each ontology; BP, Biological Process; CC, Cellular Component; MF, Molecular Function; (**B**), Distinct GO terms of the Biological Process of genes targeted by conserved and unconserved DEMs; (**C**), Venn analysis of targeted genes by conserved and unconserved DEMs.

The total 5802 target genes by DEMs were predicted with an average of ~52.0 targets per miRNA, using psRNATarget. Gene Ontology (GO) enrichment analyses showed that 3423 targets were assigned to at least one GO term and covered a broad range of 67 GO categories (Table S3). In the Biological Process, cellular process (GO:0009987), primary metabolic process (GO:0044238), cellular metabolic process (GO:0044237), macromolecule metabolic process (GO:0043170) and cellular macromolecule metabolic process (GO:0044260), represented the major groups (Figure 2A). In the Cellular Component category, membrane-bounded organelle (GO:0043227), intracellular membrane-bounded organelle (GO:0043231), nucleus (GO:0005634) and organelle part (GO:0044422) were predominant (Figure 2A). Regarding the Molecular Function category, binding (GO:0005488), transferase activity (GO:0016740) and ion binding (GO:0043167) were highly represented (Figure 2A).

The Biological process of the targeted genes by the 67 known DEMs was considered in detail. The targets of 34 conserved DEMs were assigned to 38 GO terms, including programmed cell death (GO:0012501), apoptosis (GO:0006915), death (GO:0016265) and cell death (GO:0008219) (Figure 2B, Table S4). However, only 4 GOs terms were confined to the targets of 33 unconserved DEMs, such as transport (GO:0006810), establishment of localization (GO:0051234), localization (GO:0051179) and response to stimulus (GO:0050896) (Figure 2B, Table S4). They shared the common term GO:0050896 (response to stimulus). Furthermore, Venn analysis indicated that only 42 genes were simultaneously targeted by conserved and unconserved DEMs (Figure 2C). It was indicated that the known conserved and unconserved DEMs might affect the inflorescence through a different or specific pathway. 

### 2.3. Dynamic Expression of miRNAs during Early Rice Inflorescence Development

**Figure 3:**
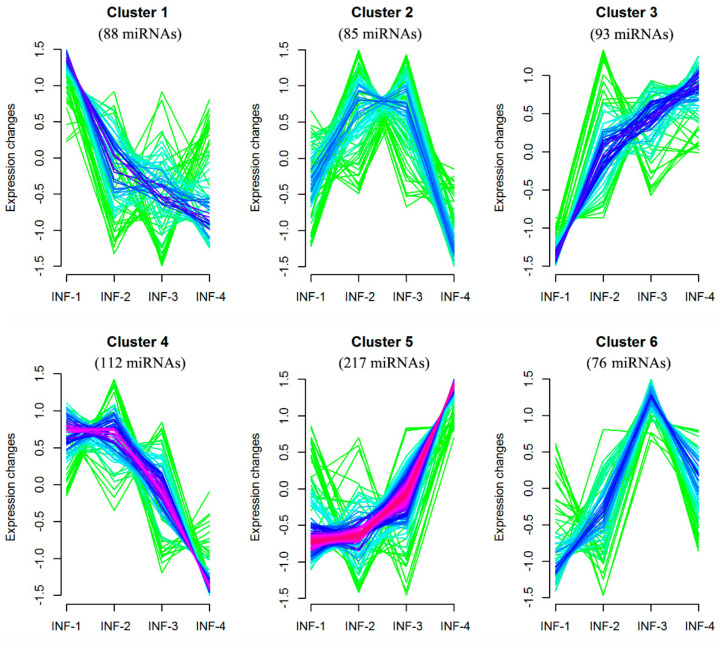
Common patterns of miRNAs expression in early rice inflorescences development.

To detect common expression patterns, TPMs of miRNAs were used to cluster using the fuzzy c-means algorithm. Six common patterns were recovered (Figure 3, Table S2). Clusters 1 and 3 displayed the opposite pattern of miRNA expression. miRNAs remained at a stable expression with high levels between INF-1 and INF-2 and then declined dramatically in Cluster 4. In contrast, miRNAs were expressed with a low level between INF-1 and INF-2, and then increased rapidly in Cluster 5. Clusters 2 and 6 demonstrated a drop of expression of miRNAs after entering the INF-3 stages. With regard to the composition of miRNAs with different lengths, the number of 40, 52, 57 and 158 21nt-length miRNAs were detected in Clusters 1, 3, 4, 5, respectively. It was indicated that the number of 21nt miRNAs with gradually increased expression was much more than those with gradually decreased expression. The ratio of 24nt miRNAs to 21nt ones was higher in Cluster 6 than in other Clusters. 

Surprisingly, the members of the conserved miRNAs family, which affects floral development, were dispersed in different Clusters. For instance, fifteen members of the osa-miR156 family were grouped into Cluster 1, 3 or 5, four of osa-miR172 into 1 or 3 and twelve of osa-miR396 into Cluster 3 or 5, while all members of osa-miR2275, osa-miR397 and osa-miR528 were listed in Cluster 5. It was surprising that 110 out of all 111 DEMs belonged to Cluster 5, except only one miRNA (osa-miR2118o) to Cluster 3. Among those known DEMs in Cluster 5, 33 members were identified as unconserved and rice-specific (Figure S2). By contrast, 22 out of these 33 rice-specific known DEMs were expressed differentially between adjacent stages during male or female gametophyte development [37], and 19 during ovule development [38], respectively. Meanwhile, 15 rice-specific DEMs, including osa-miR5506, osa-miR5516a and osa-miR5519, were commonly detected in both studies [37,38]. It was suggested that these specific DEMs might be involved in regulating the development of inflorescence, as well as the reproductive process.

### 2.4. Floral Organs Alteration in the Transgenic Lines, Overexpressing Pre-miR5506

**Figure 4:**
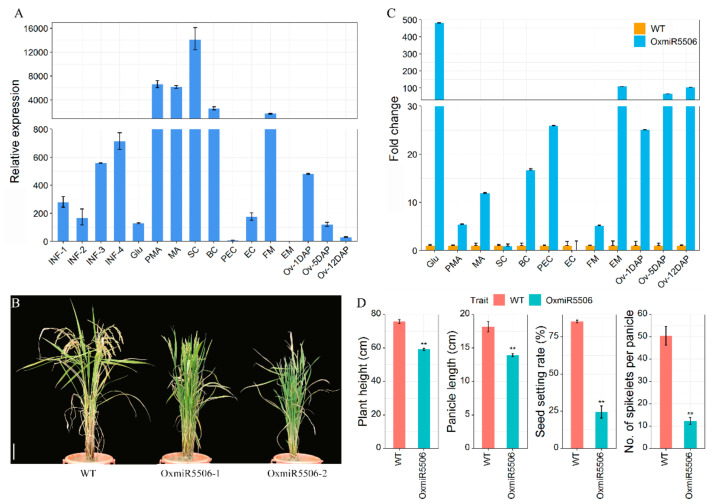
The expression pattern of osa-miR5506 and change of yield-related traits of the transgenic line, overexpressing osa-miR5506. (**A**), the expression pattern of osa-miR5506; (**B**), plant phenotype of WT and transgenic line, bar = 10 cm; (**C**), fold change of osa-miR5506 in the transgenic line OxmiR5506-1, compared to WT; (**D**), the alternation of yield-related traits of transgenic line OxmiR5506-1. WT, wild-type; OxmiR5506, the transgenic line, overexpressing pre-mature osa-miR5506; Glu, Glume; PMA, anther at pre-meiotic interphase; MA, anther at meiosis; SC, anther at single microspore stage; BC, anther at bi-cellular pollen stage; PEC, ovary at pre-meiotic interphase; EC, ovary at meiosis, FM, ovary at single microspore stage; EM, ovary at bi-cellular pollen stage; Ov-1DAP, Ov-5DAP, Ov-12DAP indicated the ovary 1, 5, 12 days after pollination, respectively; **, significant difference at 1% probability.

During evolution, some species-specific miRNAs acquired specialized functions, while their functional analysis remains to be studied [39]. osa-miR5506 were selected to experimentally validate the functions of these rice-specific DEMs. The spatio-temporal expression pattern analysis showed that the expression of osa-miR5506 was increased gradually with the process of rice inflorescence development (Figure 4A). The transcriptional level of osa-miR5506 was enhanced significantly in anther at pre-meiotic interphase (PMA) and reached the peak at the single microspore stage (SC) (Figure 4A). After pollination, osa-miR5506 was expressed at a low level and declined during the grain-filling stage (Figure 4A). To clarify the role of osa-miR5506 in the rice inflorescence development, we generated the transgenic lines (OxmiR5506), overexpressing pre-miR5506 (Figure 4B). The expression levels of os-miR5506 were significantly higher at various organs of the OxmiR5506 lines than those of wild-type rice (Figure 4C). During the vegetative stage, no obvious alteration of plant phenotype was observed in OxmiR5506 lines. At the mature stage, the plant height, panicle length, the number of spikelets per panicle and the seed set were lower in OxmiR5506 lines than those in wild-type rice (Figure 4D). It was suggested that the increased level of osa-miR5506 affected the number and fertility of spikelets. 

**Figure 5:**
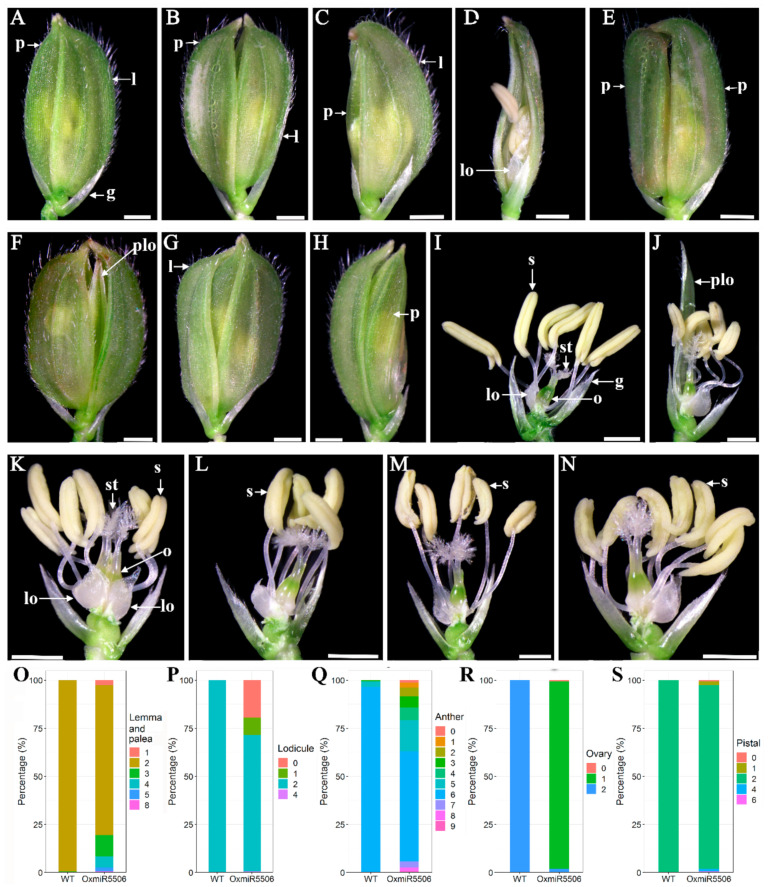
The abnormalities of floral organs in spiketlets of the transgenic line, overexpressing osa-miR5506. (**A**,**I**), the normal phenotype and number of floral organs in both WT and transgenic plants; (**B**–**H**), OxmiR5506 spikelets; (**J**–**N**), OxmiR5506 spikelets with lemma and palea removed; (**O**–**S**), percentage of various numbers of lemmas/paleas, lodicules, anthers, ovaries and pistals, respectively; g, glume; l, lemma; p, palea; lo, lodicule; plo, palea-/lemma-like organ; s, stamen; o, ovary; st, stigma; WT, wild-type; OxmiR5506, the transgenic line, overexpressing osa-miR5506; Bar = 1 mm.

The normal spikelet contains a pair of sterile glumes at the base and one floret, including lemma, palea, a pair of lodicules adjacent to lemma, six stamens and one pistil, in wild-type rice and OxmiR5506 lines (Figure 5A,I). Besides, the abnormality of spikelets was found in OxmiR5506 lines. The floret remained open due to the overdeveloped palea (Figure 5B,F). The floret with the underdeveloped palea was observed (Figure 5C). Especially, some florets lacked palea (Figure 5D). The two opposite lemmas were formed (Figure 5E). The additional pale or lemma-like organ was investigated in some spikelets (Figure 5G,H). The spikelet of the OxmiR5506 lines contained one or two pairs of lodicules (Figure 5K,L). Some lodicules were overdeveloped as a pale green or lemma-like organ (Figure 5F,J). The number of stamens varied from four to seven (Figure 5K–N). Most spikelets generated one normal ovary with two stigmas (Figure 5J,L), and the two fused ovaries with four stigmas were observed, too (Figure 5K). Furthermore, the number of abnormal floral organs of each spikelet was investigated in detail. The spikelets with overdeveloped or underdeveloped paleas constituted 5.84 and 13.87 percent of the total spikelets, respectively. A small portion of spikelets contained a various number of floral organs, such as lemmas/paleas, lodicules, anthers, ovaries and pistals (Figure 5O–S). It was indicated that the increased expression level of osa-miR5506 contributed to the incomplete determinacy of floral meristem and abnormal initiation of floral organ meristem.

### 2.5. The Abnormal Development of the Embryo Sac in the OxmiR5506 Transgenic Lines

**Figure 6:**
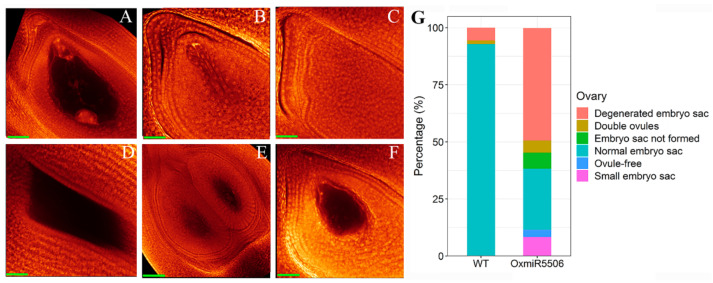
The abnormalities of mature embryo sac in OxmiR5506 lines. (**A**), normal embryo sac; (**B**), degeneration of embryo sac; (**C**), absence of embryo sac; (**D**), ovule-free ovary; (**E**), two fused ovules; (**F**), small embryo sac; (**G**), percentage of normal varies with normal embryo sac and abnormal ovaries with degenerated embryo sac, double ovules, embryo sac not formed, ovule-free or small embryo sac; WT, wild-type; OxmiR5506, the transgenic line, overexpressing osa-miR5506; Bar = 40 μm.

To explore the reason for the low seed set in OxmiR5506 lines, the fertility of mature pollen and mature embryo sacs were observed. The fertility of mature pollen in OxmiR5506 lines (75.45%) was lower than that in wild-type rice (93.49%). WE-CLSM analysis showed that the normal mature embryo sac was characterized by one egg, two synergids, two polar nucleuses and three antipodal cells in both wild-type and OxmiR5506 lines (Figure 6A). Besides, the different types of abnormalities of ovaries were found in the OxmiR5506 lines, including the degeneration of the embryo sac (Figure 6B), the absence of the embryo sac (Figure 6C), ovule-free ovary (Figure 6D), double embryo sacs (Figure 6E) and a small embryo sac (Figure 6F). The percentage of abnormal ovaries amounted to 73.27%, of which the degeneration of the embryo sac was the major type, followed by a small embryo sac and the absence of the embryo sac (Figure 6G). It was indicted that a high frequency of abnormal mature embryo sacs contributed to the low seed set in OxmiR5506 lines.

**Figure 7:**
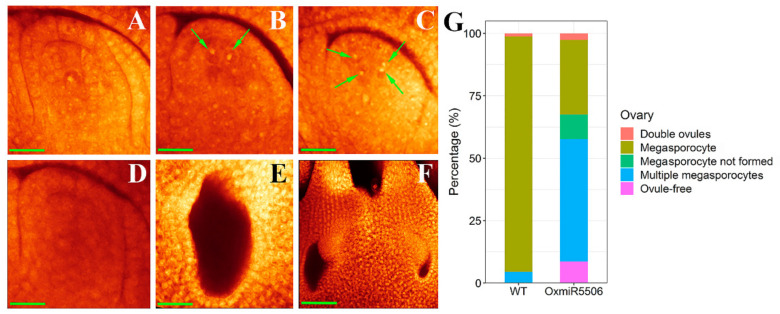
The abnormalities of ovaries on the early stage of megasporogenesis in OxmiR5506 lines. (**A**), normal ovary with a large megasporocyte; (**B**,**C**), abnormal ovary with two or four large megasporocytes, respectively; green arrow indicates megasporocyte; (**D**), abnormal megasporocyte without megasporocyte formed; (**E**), ovule-free ovary; (**F**), two fused ovaries; (**G**), percentage of normal varies with a megasporocyte and abnormal ovaries with double ovules, megasporocyte not formed, multiple megasporocyte or ovule-free; WT, wild-type; OxmiR5506, the transgenic line, overexpressing osa-miR5506; Bar = 40 μm.

To further verify the cause of the high frequency of abnormal mature embryo sacs of the OxmiR5506 lines, WE-CLSM analysis was carried out with ovaries on the early stage of megasporogenesis. In the early development of the embryo sac, only one large megasporocyte was initiated under the nucellus epidermis (Figure 7A) in both wild-type and OxmiR5506 lines. However, several abnormalities were observed in the OxmiR5506 lines, including the two or more megasporocytes (Figure 7B,C), the absence of megasporocyte (Figure 7D), ovule-free ovary (Figure 7E) and two fused ovaries (Figure 7F). The percentage of abnormal ovaries statistically was 70.20%, of which the ovaries with multiple megasporocyte constituted the majority, followed by the absence of megasporocyte and ovule-free ovaries (Figure 7G). It was indicated that OsmiR5506 exerts its role at the early stage of megasporogenesis and affects the initiation of ovule, too. 

### 2.6. Gene Expression Profile Change during Meiosis in the OxmiR5506 Lines

Cytological observation indicated that the abnormality of the embryo sac occurred during the early stage of megasporogenesis of the OxmiR5506 lines. RNA-seq was carried out to investigate the molecular effect on the abnormal phenotype of the embryo sac in the OxmiR5506 lines. For six samples, the average of raw and clean reads amounted to 46,920,794 and 46,371,725, respectively, and 91.24–96.32% of the clean reads were mapped to the rice genome (Table S5). These data indicated the sufficient read density for the quantitative gene expression analysis. A total of 6271 differentially expressed genes (DEGs) were identified, including 3182 up-regulated and 3089 down-regulated. GO enrichment analysis indicated a total of 287 GO terms were assigned to 3978 DEGs, including 2124 up-regulated DEGs related to 254 GO terms and 1800 down-regulated involved in 251 GO terms. The top 10 GO terms of Biological Process, Cellular Component, Molecular Function and up- or down-regulated genes are listed in Figure S3, respectively. In order to evaluate the accuracy of RNA-seq, the seven genes were randomly chosen using quantitative RT-PCR. The results showed that the expression pattern of seven genes was basically consistent with that determined by RNA-seq (Figure S4), confirming the reliability of the RNA-seq data. Overall, it was suggested that the obvious difference in gene expression level occurred in the transgenic line, compared to the wild-type rice. 

**Figure 8:**
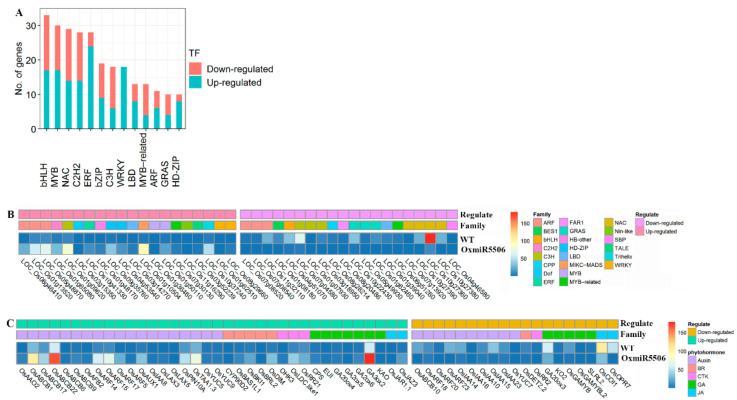
The change of expression of transcription factors and phytohormone-related genes in the OxmiR5506 lines. (**A**), the 13 gene families containing the highest number of transcription factors, which were up- or down-regulated; (**B**), the 20 transcription factors of gene families, which were mostly up- or down-regulated; (**C**), the genes involved in auxin, BR, CTK, GA and JA metabolism and signal; TF, transcription factors; BR, brassinosteroid; CTK, cytokinin; GA, gibberellin; JA, jasmonic acid.

Transcription factor (TF) and phytohormone are vital regulators of inflorescence development in rice [12]. Compared to the wild-type rice, a total of 354 TFs were regulated significantly in the OxmiR5506 lines and grouped as 44 gene families. The greatest number of DEGs TFs were found in bHLH genes family, followed by C2H2, MYB and NAC (Figure 8A). A total of 196 members of up-regulated TFs belonged to 36 gene families, and 158 that were down-regulated were classed into 37. Mostly regulated TFs were assigned to different families (Figure 8B). With respect to hormone-related genes, a total of 55 genes were significantly regulated and involved in the metabolism or signal of auxin, brassinosteroid, cytokinin, gibberellin, jasmonic acid (Figure 8C). The auxin- or gibberellin-related genes were major. It was shown that the changes of the transcription factors and hormone-related genes might be associated with the abnormalities of the development of floral organs in the OxmiR5506 lines.

### 2.7. Identification of the Target Gene of miR5506

**Figure 9:**
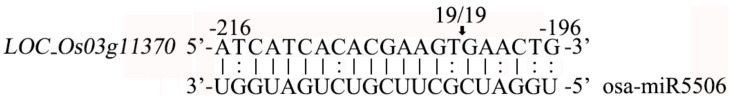
The cleavage site of target gene by osa-miR5506 confirmed with 5′–RACE. The arrow points to the osa-miR5506–directed cleavage site at *LOC_Os03g11370*.

To identify the molecular mechanism of osa-miR5506 regulating reproductive development, potential targets by osa-miR5506, including *LOC_Os03g11370*, were first predicted with psRNATarget. qRT-PCR results showed the expression of target genes *LOC_Os03g11370* were down-regulated in the OxmiR5506 lines (Figure S5). The analysis on RLM-RACE indicated that *LOC_Os03g11370* was cleavaged between the sixth and seventh base pair of the osa-miR5506 target site (Figure 9). *LOC_Os03g11370* is a member of the REM (Reproductive Meristem) transcription factor gene family. 

## 3. Discussion 

### 3.1. A Comprehensive miRNAs Analysis Uncovers Many Uncharacterized Members That Might Affect Early Inflorescence Development in Rice

The rice inflorescence undergoes a complex series of developmental events. Especially, the activity of different meristems, including branch, spikelet, floret and floral organ meristems, occurs dramatically during early young inflorescence development [40]. The molecular mechanisms underlying the early inflorescence development have been studied extensively. Numerous protein-coding genes are characterized, such as *FON1* and *FON2* involved in the CLV-WUS signal pathway [9,10], *SEP* homologs *OsMADS34*/*PAP2* acting synergistically with *LAX1*) and *FON4* [41,42], *LAX1* related to the auxin pathway [43], *OsCKX2*, *LARGER PANICLE* (*LP*), *DROUGHT AND SALT TOLERANCE* (*DST*) involved in the cytokinin signal pathway [8,44,45] and the conserved MADS-box genes [46]. Epigenetic factor osa-miR156 mediates inflorescence branching by repressing the expression of *OsSPL14* [27]. The changes of expression of protein-coding genes of different meristems or different stages during early rice inflorescences development were investigated by using microarray or RNA-seq [34,47,48,49]. Meanwhile, the expression of miRNAs expression at later stages of rice inflorescences development was carried out [35]. However, the information about the dynamic change of miRNAs expression during early inflorescence development remains to be elucidated.

In this study, the high abundance of miRNAs was exhibited in all four different stages (Figure 1A). Especially, the expression level and number of 21nt miRNAs were increased gradually with the inflorescence development, compared to the other types of miRNAs (Figure 1A). Meanwhile, the 21nt miRNAs accounted for most members of the miRNA family in each stage (Figure 1B), suggesting their important role in regulating the inflorescence development. The population of 671 miRNAs was identified in all four stages, and greater than the number of miRNAs detected in leaf, root, pollen embryo sac or ovule, respectively [37,50,51]. The increased number of miRNAs might be related to the active proliferation of meristem cells, which ultimately develop into panicle branches, spikelets, florets and floral organs. Furthermore, a total of 111 DEMs, including 67 known DEMs (Table S6), were identified, and most DEMs occurred during the transition of the latter three stages, during which the meristems of spikelets and floral organs are continuously and sequentially specified. Among DEMs, osa-miR396d affects the floral organogenesis by targeting *Growth Regulating Factor 6* (*OsGRF6*) [52], and osa-miR397b promotes panicle branching and increases grain size by regulating *Laccase* (*OsLAC*) [29]. Venn analysis indicated that 65 out of the 67 known DEMs in this study were commonly expressed in the leaf, root, pollen or embryo sac [37,53], except osa-miR5494 and osa-miR5495 specific to inflorescence. *GIBBERELLIN INSENSITIVE DWARF1* (*GID1*), a predicted target of osa-miR5495, encodes gibberellin receptor GID1L2. The *gid1-1* did not develop fertile flowers [54]. *LARGE1/OML4*, which is predicted to be targeted by osa-miR5494, regulates cell expansion in spikelet hulls [55]. It was curious to explore the regulations of DEMs in the development of early inflorescence.

### 3.2. Implication of Specific miRNAs Functioning in the Development of Early Inflorescence in Rice

In rice, the constitutively expressed conserved osa-miRNAs were involved in various aspects of biological development, such as osa-miR156, osa-miR397, osa-miR396 regulating panicle architecture and osa-miR172, osa-miR159, osa-mi529 affecting inflorescence development [25]. Especially, osa-miR528 exerts pleiotropic roles in flowering time, antiviral response, or reactive oxygen species (ROS) homeostasis [56,57,58]. The miRNA profiles were carried out with diverse organs or tissues, including root, leaf, callus and developing pollen [53], pollen and embryo sac [37], ovule [38,50] and filling grain [59,60]. In contrast to those miRNAs in the above-mentioned studies, a total of 41 miRNAs were identified as inflorescence-specific (Table S7). Among them, osa-miR5495 and osa-miR5494 were DEMs, but the others were not. These 41 inflorescence-specific miRNAs were expressed in developing spikelets [61]. psRNATarget analysis revealed that these 41 inflorescence-specific miRNAs targeted 1802 genes, of which 19 targeted genes, such as *OsYUCCA2,* *FRIZZLE* *PANICLE* (*FZP)*, *OsHUS1* and *OsJAZ1,* regulated the development of the floral organ or male sterility (Table S8). Using microarray, the gene expression profiles of early inflorescence development were characterized in *japonica* (RiceXPro, https://ricexpro.dna.affrc.go.jp/, accessed on 15 August 2020) and *indica* rice (CREP, http://crep.ncpgr.cn/crep-cgi/home.pl, accessed on 15 August 2020), respectively. By comparison, only 7 of 1802 targets were regarded as differentially expressed in RiceXPro and 43 were gradually changed from the early to late stage of panicle development in CREP (Table S9). It was speculated that these inflorescence-specific miRNAs might act as a kind of household-like regulators to keep their targeted genes under proper expression levels for the proper development of inflorescence. 

In contrast to the conserved miRNAs functioning in various aspects of plant development, only a few rice-specific miRNAs were functionally characterized, such as osa-miR1848, osa-miR1873 or osa-miR7695 involved in phytohormone synthesis [62,63,64]. Among common patterns of miRNAs expression, Cluster 5 contained 110 DEMs, which included 33 rice-specific known DEMs. In contrast with the DEMs detected in male and female gametophyte development [37] and ovule development [38], 15 out of 33 rice-specific DEMs, including osa-miR5506, osa-miR5516a and osa-miR5519, were commonly shared (Figure S2). It is suggested that these specific DEMs might be involved in regulating the development of inflorescence, as well as the reproductive process.

### 3.3. Rice-Specific osa-miR5506 Regulates the Development of Floral Organs and Megagametophyte 

Many conserved miRNAs were characterized to regulate floral development, such as osa-miR156, osa-miR172, osa-miR396 and osa-miR397. In this study, we uncovered that osa-miR5506 affected the floral organ development and floral meristem determinacy by over-expressing precursor osa-miR5506. The abnormalities mainly including overdeveloped/underdeveloped paleas, a changed number of floral organs, fused ovaries and- adecreased number of spikelets per panicle. In rice, the alternation of size, maintenance and determinacy of floral meristem would cause defects in floral development. *FON2 SPARE1* (*FOS2*) redundantly regulates floral meristem maintenance with *FON2* in rice [65]. *SPW1*/*OsMADS16* is a B-class homeotic gene and interacts with *OsMADS3* and *OsMADS58* in specifying floral patterning [66]. The expression levels of several genes involved in floral development, such as *SPW1*/*OsMADS16*, *OsMADS34*, *FOS1*, *OSH1* and *ABERRANT PANICLE ORGANIZATION 2* (*APO2*), were regulated differentially in the transgenic lines by RNA-seq, basically elucidating the cause for abnormal development of floret. It was indicated that osa-miR5506 might exert important roles in the development of floral meristem.

With respect to the low seed set in the transgenic lines, the fertility of mature pollen and embryo sac was examined. The high percentage of abnormalities of ovaries (73.27%) corresponded well with the low seed set in OxmiR5506 lines (Figure 4 and Figure 6). The degeneration of the embryo sac, the absence of the embryo sac, ovule-free, double embryo sacs, and a small embryo sac were the main types of abnormalities of ovaries. The 70.20% of abnormal ovaries development during the early stage of megasporogenesis provided substantial evidence for the low seed set. The abnormal phenotype of multiple megasporocytes was about half (49.01%) of the total ovaries observed. The presence of multiple megasporocytes was found in *a TPD1-like gene in rice* (*OsTDL1A*)*-RNAi* and *msp1* plant; OsTDL1A binds MSP1 to limit sporocyte numbers [16,67]. The expression level of *OsTDL1A* and *MSP1* was regulated significantly in the OxmiR5506 lines. The defects of double ovules or ovule-free ovaries were identified in the OxmiR5506 lines, too. The down-regulation of *Indeterminate Gametophyte1* (*OsIG1*) results in unusual double ovules and developmental abnormalities of various floral organs and megagametophytes [68]. However, *OsIG1* was up-regulated in the OxmiR5506 transgenic line. The ovule-free ovaries were found in the mutation of *OsPINOID*, which was suggested to regulate stigma and ovule initiation by auxin signaling [69]. *AtARF6* and *AtARF8* are members of the plant-specific B3 superfamily and mutations in the target sites by AtmiR167 caused the growth of ovule integuments arrested, and anthers grew abnormally [70]. In this study, the 5′-UTR of *LOC_Os03g11370*, a member of the REM sub-family that belongs to the plant-specific B3 superfamily, was cleavaged by osa-miR5506 (Figure 9). The altered expression level of *Oryza sativa REPRODUCTIVE MERISTEM 20* (*OsREM20*) contributes to the variation of the grain number per panicle among rice varieties, and OsMADS34 directly binds the CArG box within the *OsREM20* promoter [71]. The MADS-box gene *SEEDSTICK* (*STK*), *SHATTERPROOF1* (*SHP1*) and *SHP2* directly target the CArG boxes of the promoter of the REM gene *VERDANDI*, which affects embryo sac differentiation in *Arabidopsis* [72]. It was suggested that osa-miR5506 regulates the development of megagametophyte in rice, possibly through targeting *LOC_Os03g11370*, which might be regulated by the MADS-box gene.

## 4. Materials and Methods

### 4.1. Plant Materials

The *japonica* cultivar Nipponbare (*Oryza sativa* L.) was grown in an experimental field at the South China Agriculture University (Guangzhou, China) under field conditions. According to the stage development by Sharma et al. [73] and minor modification, a total of 12 RNA samples were prepared from young inflorescence at four developmental stages, including young inflorescences with less than 2 mm length (INF-1), 2–5 mm length (INF-2), 5–10 mm length (INF-3) or 11–15 mm length (INF-4), with three biological replicates. All the samples were kept at −80 °C for RNA-seq, the expression pattern of osa-miR5506 and target genes. 

### 4.2. Analysis of the Profile of miRNAs

Total RNA was extracted with TRIzol Reagent (Invitrogen, Waltham, MA, USA). Small RNA libraries construction was conducted with Next Ultra small RNA Sample Library Prep Kit (NEB) and then was sequenced using Illumina sequencing (HiSeq2500). Raw data were subjected to quality filtering with removal of low-quality reads, adapter sequence and the reads containing over 10% of unknown bases. The reads less than 18 bp or over 30 bp nucleotide were discarded from the total reads. With Bowtie software, the clean reads were used to blast against the database, including Silva, GtRNAdb, Rfam and Repbase, to remove rRNA, tRNA, snRNA, snoRNA and repeat sequences. The unannotated reads were applied to predict known and novel miRNAs by miRDeep2. The miRNA expression of transcript in the samples was normalized to obtain the expression of transcript per million (TPM). The differentially expressed miRNAs (DEMs) were identified with DESeq software with a criterion (|log2(FC)| ≥ 1, FDR ≤ 0.01). Target genes of miRNAs were predicted using psRNATarget with the default parameters [74]. The agriGO online serve was used to annotate the function of target genes (http://systemsbiology.cau.edu.cn/agriGOv2/index.php, accessed on 15 August 2020). 

### 4.3. qRT-PCR Analysis of miRNAs and Targeted Genes

The expression profile of mature miRNAs and their target genes was validated using the qRT-PCR technique. The total RNA was used to synthesize reverse transcripts using the One Step PrimeScript miRNA cDNA Synthesis Kit (Takara, Japan) in a 10 μL reaction mixture. The reaction was performed at 37 °C for 1 h and 85 °C for 5 min for the reverse transcription. The primers were designed by the Primer premier 5.0 software (Table S10). Quantitative real-time PCR (qRT-PCR) analysis was performed with LightCycler 480. A total of 20 μL SYBRH Premix Ex TaqII (Takara, Japan) reaction mixture contained 10 μL SYBR Premix Ex Taq (Tli RNaseH Plus), 0.4 μL each primer (10 μM) and 2 μL cDNA. The PCR amplification procedure was set to incubate at 95 °C for 5 s, followed by 40 cycles of the denaturation at 95 °C for 5 s and the annealing and extension at 60 °C for 30 s. All samples were conducted with three biological replications. The relative expression level of miRNAs was calculated using the 2^−ΔΔCT^ method [75].

### 4.4. Plasmid Construction and Plant Transformation

To overexpress osa-miR5506 in *japonica* rice Nipponbare, the genomic DNA sequence of the pre-miR5506 was amplified with specific primers (Table S10). The PCR product was cloned into *Kpn*I/*Bam*H I restriction sites of pOX binary vector between the maize *Ubiquitin* promoter and the *Nos* terminator. The transgenic rice plants were generated with *Agrobacterium*-mediated transformation [76]. The T_3_ homozygotes of transgenic lines were selected for further study.

### 4.5. Analysis of Agronomic Traits and Cytological Observation of Embryo Sac Development

The agronomic traits, including plant height, the number of tillers, panicle length, the number of spikelets per panicle, seed set rate, grain length, grain width and 1000-grain weight, were examined in the transgenic lines. Student’s *t* test was used to detect the significance of the difference in agronomic traits between WT and transgenic plants. The ovaries tissues were collected at the early stage of megasporogenesis and mature stages, respectively, and fixed in FAA solution (50% ethanol:acetic acid:acetaldehyde = 89:6:5) for 48 h, washed with 50% ethanol and kept in 70% ethanol at 4 °C. A whole mount eosin B confocal laser scanning microscopy (WE-CLSM) was applied to observe the development of the embryo sac, following the previous protocol [77].

### 4.6. RNA-Seq Library Construction, Sequencing and Analysis

The ovary tissues were collected at the early stage of megasporogenesis stages with three biological replications. Total RNA of each sample was extracted with TRIzol Reagent (Invitrogen), quantified and qualified by Agilent 2100 Bioanalyzer (Agilent Technologies, Palo Alto, CA, USA), NanoDrop (Thermo Fisher Scientific Inc, MA, USA.) and agarose gel. The RNA with a RIN value above 6.5 was used for following library preparation. The sequencing library preparations were constructed according to the manufacturer’s protocol. Sequencing was carried out using a 2 × 150 bp paired-end (PE) configuration at the GENEWIZ, Inc. (Suzhou, China). 

For quality control, the raw reads with fastq format were collected from all samples. The high-quality clean reads were generated by Cutadapt (V1.9.1) software by removing adapters sequences, polymerase chain reaction (PCR) primers and quality of bases lower than 20. The clean reads were mapped to the rice reference genome (MSU7.0, http://rice.plantbiology.msu.edu/index.shtml, accessed on 18 September 2019) via Hisat2 (v2.0.1) software. Then, the expression levels of genes were estimated from the pair-end clean data with Cuffdiff (v2.2.1). The differentially expressed genes (DEGs) were defined according to the criterion of |log2 (fold change)| ≥ 1 and *q* value ≤ 0.05. The gene ontology (GO) annotations of DEGs were obtained using GOseq method. The qRT-PCR was applied to validate the reliability of RNA-seq with the primer listed in Table S10.

### 4.7. RLM-RACE

To detect the cleavage sites of the target gene by OsmiR5506, 5ʹ-RACE cDNA was generated using the SMARTer^TM^ RACE cDNA Amplification Kit (Clontech). Two 5ʹ-RACE gene-specific primers were designed for nested PCR. The first cycle of PCR was performed with 5ʹ-RACE cDNA as template and the specific primer 5′-CCTAAACACACACACACACGACGAGACG-3′. Then, the PCR products were applied as template for the second cycle of PCR with the specific primer 5′-TCCTGGAACTCCATCGGCACGTTCAAG-3′, using the KOD-Plus-Neo (TOYOBO). Finally, the PCR products with the predicted size were sequenced.

## Data Availability

RNA sequencing raw data have been deposited to National Center for Biotechnology Information (NCBI) under accession numbers SUB9604963 and SUB9604976.

## Supplementary Materials

The following are available online at https://www.mdpi.com/article/10.3390/ijms22126610/s1.
