# Supplementary material for: MircroRNA Profiles of Early Rice Inflorescence Revealed a Specific miRNA5506 Regulating Development of Floral Organs and Female Megagametophyte in Rice
Source: Int J Mol Sci. 2021 Jun 21;22(12):6610. doi: 10.3390/ijms22126610 (PMC8235126; doi:10.3390/ijms22126610)
Supplement: Supplementary file 1 [file ijms-22-06610-s001.zip › ijms-1236069-SUP-PROOF DONE/Supplemental material/Supplemental Figures S1-S5.pdf]

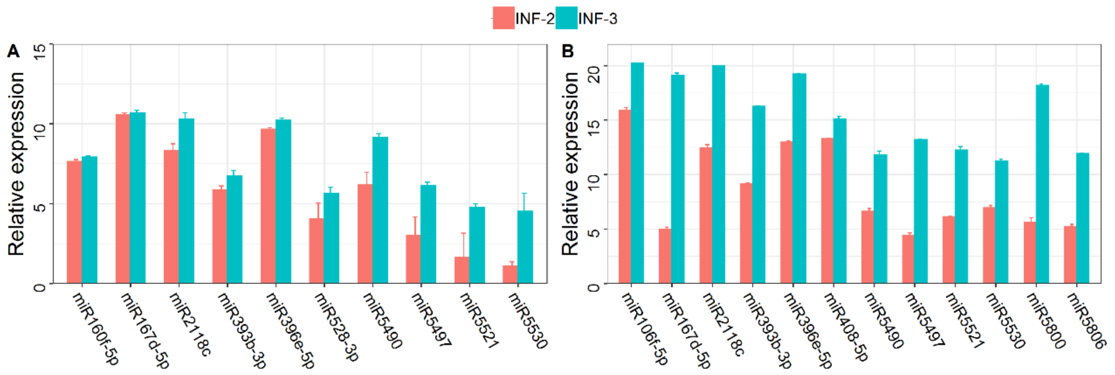

Figure S1. QRT–PCR validation of miRNA sequence of early rice inflorescence development

A, miRNA seq; B, qRT-PCR.

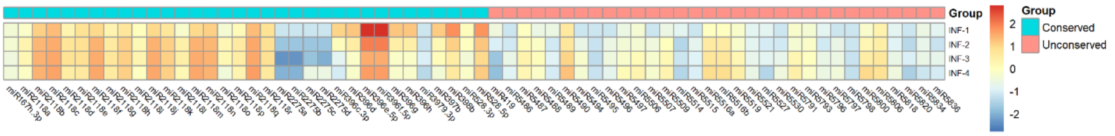

Figure S2. Heatmap cluster of the conserved and unconserved DEMs

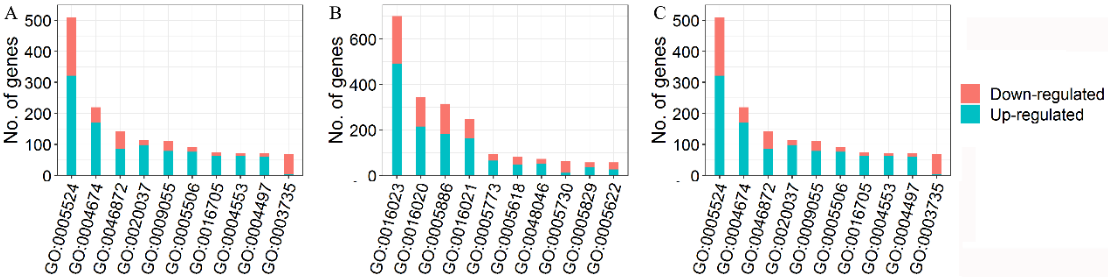

Figure S3. The top 10 GO terms of up- or down-regulated genes in the OxmiR5506 transgenic line

A, Biological Process; B, Cellular Component; C, Molecular Function.

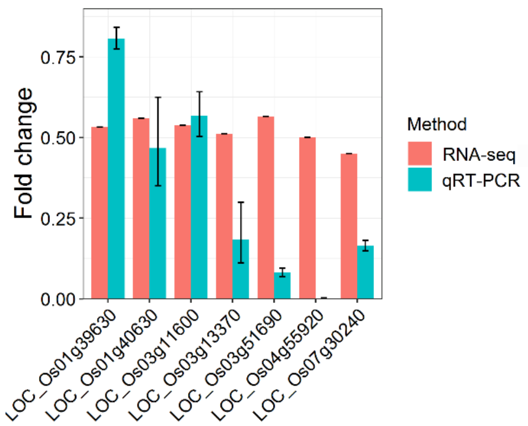

Figure S4. QRT-PCR validation of RNA-seq of the OxmiR5506 transgenic line

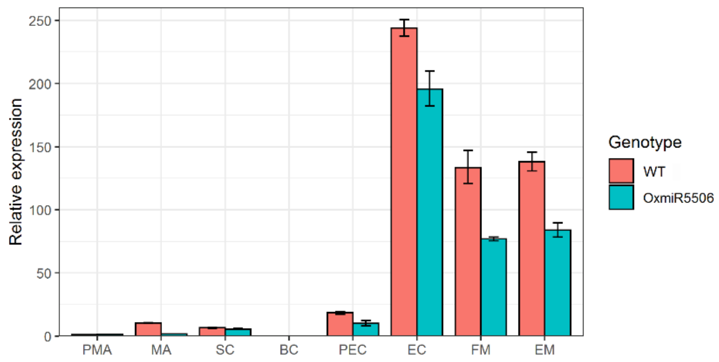

Figure S5 Target gene LOC_Os0g11370 down-regulated in the OxmiR5506 transgenic lines

WT, wild-type; OxmiR5506, the transgenic line, overexpressing osa-miR5506; PMA, anther at pre-meiotic interphase; MA, anther at meiosis; SC, anther at single microspore stage; BC, anther at bi-cellular pollen stage; PEC, ovary at pre-meiotic interphase; EC, ovary at meiosis, FM, ovary at single microspore stage; EM, ovary at bi-cellular pollen stage.
